# Social robots as effective language tutors for children: empirical evidence from neuroscience

**DOI:** 10.3389/fnbot.2023.1260999

**Published:** 2023-11-27

**Authors:** Maryam Alimardani, Jesse Duret, Anne-Lise Jouen, Kazuo Hiraki

**Affiliations:** ^1^Department of Cognitive Science and Artificial Intelligence, Tilburg University, Tilburg, Netherlands; ^2^Department of General Systems Studies, The University of Tokyo, Tokyo, Japan; ^3^INSERM UMR1093-CAPS, UFR des Sciences du Sport, Université Bourgogne Franche-Comté, Dijon, France

**Keywords:** child-robot interaction (CRI), robot-assisted language learning (RALL), electroencephalography (EEG), functional connectivity (FC), Phase-Locking Value (PLV)

## Abstract

The aim of the current study was to investigate children's brain responses to robot-assisted language learning. EEG brain signals were collected from 41 Japanese children who learned French vocabularies in two groups; half of the children learned new words from a social robot that narrated a story in French using animations on a computer screen (Robot group) and the other half watched the same animated story on the screen but only with a voiceover narration and without the robot (Display group). To examine brain activation during the learning phase, we extracted EEG functional connectivity (FC) which is defined as the rhythmic synchronization of signals recorded from different brain areas. The results indicated significantly higher global synchronization of brain signals in the theta frequency band in the Robot group during the learning phase. Closer inspection of intra-hemispheric and inter-hemispheric connections revealed that children who learned a new language from the robot experienced a stronger theta-band EEG synchronization in inter-hemispheric connections, which has been previously associated with success in second language learning in the neuroscientific literature. Additionally, using a multiple linear regression analysis, it was found that theta-band FC and group assignment were significant predictors of children's language learning with the Robot group scoring higher in the post-interaction word recognition test. These findings provide novel neuroscientific evidence for the effectiveness of social robots as second language tutors for children.

## 1 Introduction

Social robots have the potential to change the landscape of education as they are being progressively integrated into learning environments (Belpaeme et al., 2018a; Van den Berghe et al., 2019; Johal, 2020; Woo et al., 2021). Multiple studies have demonstrated the effectiveness of humanoid social robots in promoting language learning among children; an application domain known as Robot-Assisted Language Learning (RALL) (Randall, 2019; Van den Berghe et al., 2019; Lee and Lee, 2022). In RALL studies, a social robot usually takes the role of a tutor (Vogt et al., 2019) or a peer (Mazzoni and Benvenuti, 2015) in one-on-one or group-based learning interactions to teach children new vocabulary and expressions in a second language (Randall, 2019). These studies often argue that robots can be highly effective language instructors, particularly for young children as opposed to adult learners (Lee and Lee, 2022), because of their physical embodiment and social behavior that not only increases engagement and motivation in this particular user group, but also facilitates natural communication through non-verbal gestures that ultimately improve learning gain among children (de Wit et al., 2018; Schodde et al., 2019).

However, previous Child-Robot Interaction (CRI) studies have predominantly employed self-reported questionnaires from children (Kennedy et al., 2016), behavioral measures of task engagement extracted from video recordings (Vogt et al., 2019; De Haas et al., 2020; Lytridis et al., 2020) and post-interaction vocabulary tests (De Haas et al., 2020; Alimardani et al., 2021) to assess the children's experience and learning gain during RALL. Although questionnaires offer insights into a child's perception of the robot, they are not always ideal as preschoolers cannot fill out surveys on their own and their response might be influenced by the presence of an experimenter. On the other hand, analysis of children's behavior from video recordings requires considerable effort from human annotators and while post-interaction language tests provide a direct assessment of the learning performance, they do not account for individual differences in the learning process.

An alternative approach to subjective and behavioral measures, is the usage of neuroscientific methods to quantify brain responses during CRI. For instance, using electroencephalography (EEG), Alimardani et al. (2021) showed that the presence of a social robot could induce higher levels of engagement in children's brain signals as quantified by frequency-domain features in the central regions of their brain. In another study (Goulart et al., 2019), EEG signals were collected from a group of children, diagnosed with Autism Spectrum Disorder (ASD), who interacted with a social robot. The study found a more pronounced beta-band activity in the children's frontal brain region when they interacted with the robot, which was presented as evidence for activation of language and social behavior functions that are usually impaired in this group of children. Although only tested with adult population, EEG brain signals have also been employed in human-robot interaction studies to measure user attention in a learning context (Szafir and Mutlu, 2012; Kompatsiari et al., 2018; Charpentier et al., 2022; Vrins et al., 2022).

Among non-invasive neuroimaging techniques, EEG provides a portable, temporally accurate and cost-effective method for research into neural processes and hence is considered as the most practical tool for measuring brain activity changes of children while they engage in a learning task (Xu and Zhong, 2018). Particularly in the context of second language learning, the CRI field can take inspirations from past studies that have examined EEG brain patterns of children associated with language production and comprehension tasks (Maguire and Abel, 2013; Gaudet et al., 2020) to investigate the impact of technology-assisted learning on children's brain.

When discussing neuroscience of language learning, the traditional view holds that language processing is lateralized in the left hemisphere, however, recent research suggests that the right hemisphere also plays a critical role in successful language acquisition and hence a more distributed network is activated particularly in the early stages of second language learning (see the review by Qi and Legault, 2020). One of the measures that is often employed in neuroscientific literature as an indicator of language development is functional connectivity (FC), which refers to the degree of synchronization between different brain regions as a consequence of their interaction and communication (Gaudet et al., 2020; Yoon et al., 2021). In a systematic review of EEG-based functional connectivity reports related to language functions, Gaudet et al. (2020) observed that theta band oscillations were associated with language development and that a larger FC across brain regions in the theta band was indicative of better language functions in early childhood. Additionally, multiple studies have demonstrated that a learner's attainment of new words is dependent on the left and right inter-hemispheric FC (Veroude et al., 2010; Gaudet et al., 2020; Sander et al., 2023), providing evidence that both hemispheres interact for a successful language acquisition.

Based on these past reports, we identified the literature gap for neuroscientifically-grounded research in the field of CRI. If social robots are effective language tutors, then they should be able to activate brain networks that have been previously identified by neuroscience literature of language acquisition (Gaudet et al., 2020). Therefore, the current study aimed to (1) measure EEG functional connectivity in children's brain activity when they learned new vocabularies in a foreign language from a social robot as opposed to a non-social technology, and (2) probe the relationship between children's brain activity changes during the learning phase and their recall of the words afterwards. Two groups of Japanese children participated in a second language learning task facilitated by different technology forms; one group watched an animated story in French language on a computer screen (Display group) and the other group watched the same animation narrated by a NAO robot that employed gestures to augment the storytelling (Robot group). The Display group served as the control group for comparison with the Robot group. EEG brain activity was recorded from both groups during the learning task and Phase-Locking Value (PLV) between all electrode pairs was extracted as a metric of FC (Leeuwis et al., 2021; Yoon et al., 2021). Additionally, children's learning performance was evaluated in a post-interaction word recognition test. We hypothesized that changes in EEG functional connectivity would be stronger among children who learned a new language from a social robot than those who learned the words from the computer screen. Additionally, we expected that the level of FC across brain regions would be related to the learning performance of children as evaluated by the post-interaction word test.

## 2 Methods

### 2.1 Participants

**Figure 1:**
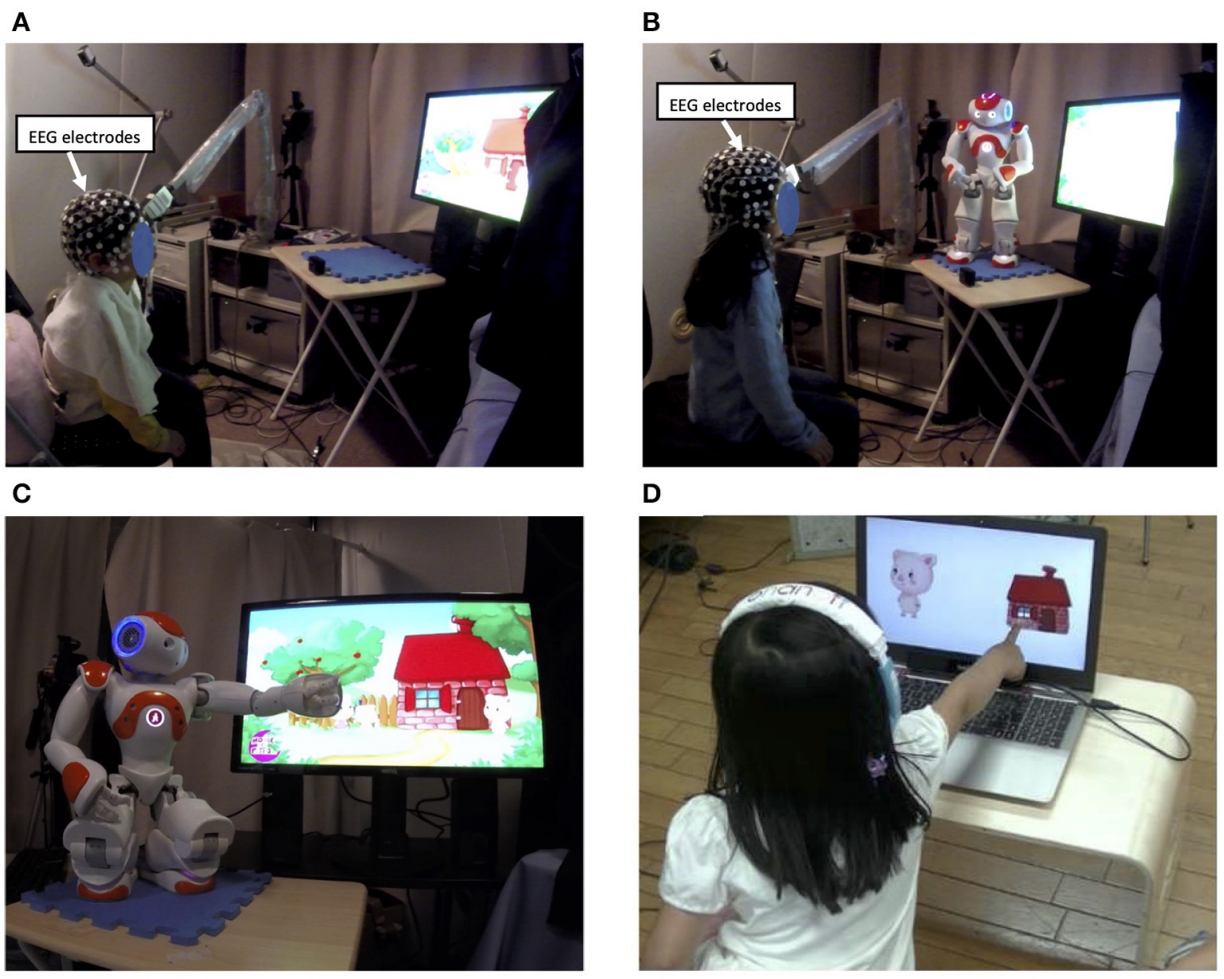
Overview of the experiment setup. Participants were divided into two groups, each experiencing one experimental condition; **(A)** the Display group watched an animated story with a pre-recorded narration in French on a computer screen; **(B)** the Robot group watched the same animation, but the story was narrated by a NAO robot; **(C)** the robot performed pointing or iconic gestures whenever a target word (e.g., la maison meaning a house) appeared in the story; **(D)** after the interaction, word learning was assessed in both groups through a word recall test.

Forty-one Japanese children participated in this experiment (22 boys, 19 girls, *M*_age_ = 5.53, *SD*_age_ = 0.15). The children had no prior exposure to the French language. Upon admission to the study, they were randomly assigned to one of the experimental groups: Display group (n=21) or Robot group (n=20). Children in the Display group listened to an animated story in French that was displayed on a computer screen in front of them (Figure 1A). The other group watched the same animation on the screen but listened to the narration of the story by a NAO robot that gestured toward the screen whenever the target word appeared in the animation (Figure 1B). The study was approved by the Ethics Committee of the University of Tokyo. Before the experiment started, the parents of the children received information about the study and signed a written consent form.

### 2.2 Experiment procedure

After children received instructions about the task, they were seated in front of a computer screen in a shielded experiment room. The experimenter adjusted the EEG cap and checked the electrode-scalp impedance to ensure good contact and sufficient signal quality.

The entire recording took about 7 mins. Before the learning phase started, there was an introduction phase of 15 seconds in which the children were greeted either by the robot or by a pre-recorded voice. Next, the learning phase started, during which children listened to an animated story in French and were expected to learn three target words (pig, house and wolf in French). For both Robot and Display groups, animated illustrations of the story were presented on the screen that matched the narrative. For instance, when the word “la maison” (i.e., French word for “house”) was verbalized, a picture of a house was shown in the animation. The only difference between the conditions was the presence of a NAO robot in the Robot condition, which narrated the story and presented a rich variety of behavior to support the storytelling. The robot was able to play audio sounds (e.g., laughs or onomatopoeia representing a pig's growl or a wolf's howling) and presented pointing or iconic gestures whenever a target word appeared on the screen. For instance, whenever the target word “la maison” appeared in the narrative, the robot pointed to the animated house on the screen (Figure 1C). Children watched the story only once but were frequently exposed to the three target words they were supposed to learn from the story.

Once the learning phase was over, the children participated in a word recall test. In this testing phase, children heard a French word in an earphone and were supposed to select the associated picture from two choices on the screen (Figure 1D). There were 24 questions in total; half of them included one of the target words tested against another target word or against a completely new word (a distracter), and the other half included images of only distractor words (e.g., duck, bear, chicken, rabbit). The percentage of all correct answers given for either target or non-target words was then obtained as an indicator of the children's learning performance.

### 2.3 EEG recording

**Figure 2:**
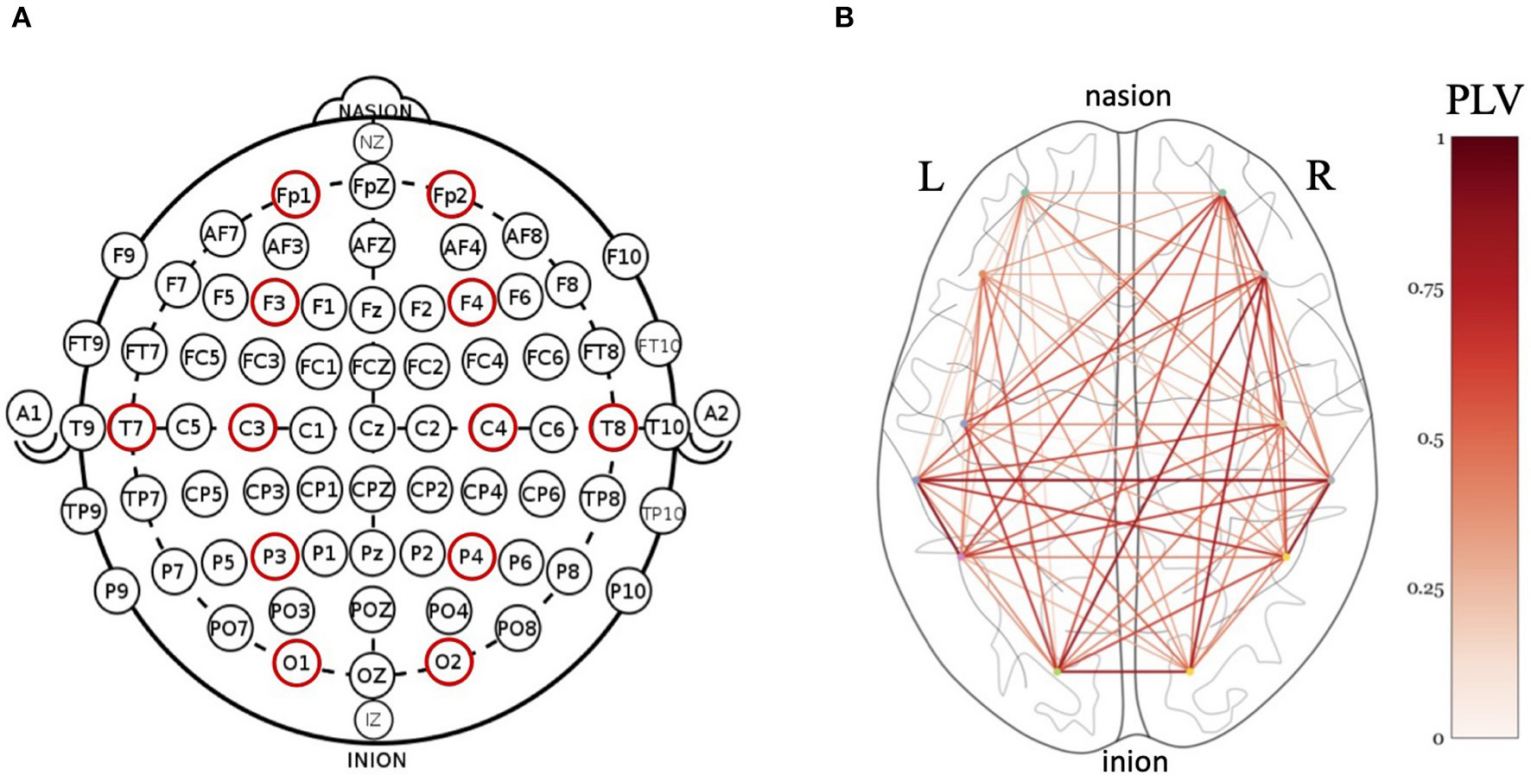
**(A)** Twelve EEG electrodes (indicated in red) covering prefrontal (Fp1 and Fp2), frontal (F3 and F4), central (C3 and C4), temporal (T7 and T8), parietal (P3 and P4), and occipital (O1 and O2) regions were selected for functional connectivity analysis. **(B)** An example illustration of PLV values during one second of interaction with the robot. Darker colors of red indicate higher PLV value and hence stronger connectivity between brain regions.

The brain signals were acquired by a 64-channel EEG cap (Electrical Geodesics Inc., US) suitable for experiments with children. The recording sampling rate was set at 250Hz. Since the central EEG electrode (Cz) was used as the reference electrode during the recording, this channel was reconstructed later via re-referencing, yielding a total of 65 channels. To reduce volume conductivity among neighboring electrodes, only 12 out of the 65 channels were selected for further analysis. These were electrodes Fp1, Fp2, F3, F4, C3, C4, T7, T8, P3, P4, O1, and O2 which cover pre-frontal, frontal, central, temporal, parietal and occipital brain regions in both hemispheres as shown in Figure 2A.

The EEG signals were pre-processed in MATLAB (version R2020b). This included manual rejection of bodily movements, removal of low/high frequency noise components using a bandpass filter of 4–30 Hz, and finally application of Independent Component Analysis (ICA) for removal of eye-blink artifacts (see details in Alimardani et al., 2021). Next, using the Hilbert Transform in the Python HyPyP library (Ayrolles et al., 2021), EEG signals were transformed into the time-frequency domain and decomposed into three frequency bands of theta (4–8 Hz), alpha (8–13 Hz) and beta (13–30 Hz) for functional connectivity analysis.

### 2.4 Functional connectivity analysis

The term functional connectivity (FC) refers to the relationship between two brain regions as a result of their interaction and shared neural patterns. There are different approaches for measuring FC from EEG oscillations that are collected from different electrode sites (Bastos and Schoffelen, 2016). For this study, we employed Phase-Locking Value (PLV), which estimates phase synchronization between two signals according to Equation 1:


(1) $$\begin{array}{l} \text{PLV}\left(\text{t}\right)=\frac{1}{N}|\overset{\text{N}}{\underset{\text{n}=1}{\sum }}e^{j\varphi \left(t\right)}| \end{array}$$

In this equation, *t* is the time interval, *N* is the number of samples and φ is the phase difference between two signals. PLV produces values in the range of 0 (absence of synchronization) to 1 (complete phase locking) for every pair of EEG channels (see an example in Figure 2B).

**Figure 3:**
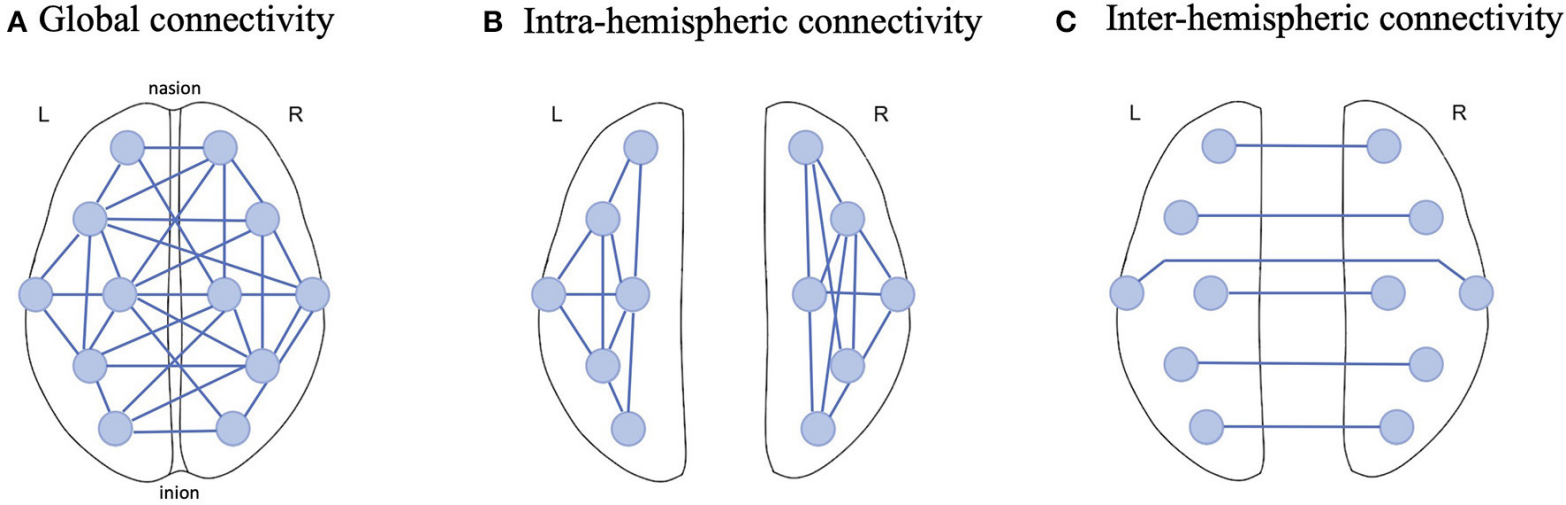
Example illustration of EEG functional connectivity scales examined in this study; **(A)** Global connectivity was obtained by computing the average PLV across all possible connections between 12 EEG electrodes, **(B)** Intra-hemispheric connectivity was computed by only averaging the connections in one hemisphere and **(C)** Inter-hemisphere connectivity was estimated by averaging the PLV across homologous electrodes in the left and right hemispheres.

The current study employed 12 EEG electrodes from the recordings and hence 66 possible pairs were established for PLV calculation in the three frequency ranges of interest (theta, alpha and beta). To aggregate these pairs, we considered 3 connectivity scales: Global, Intra-hemispheric and Inter-hemispheric connectivity (Figure 3). For Global connectivity, we took the average of PLV values from all 66 pairs as a holistic metric of connectivity across all brain regions (Figure 3A). Intra-hemispheric connectivity was obtained by averaging the PLV values across the connections in each hemisphere separately; that is 15 connections in the left hemisphere and 15 connections in the right hemisphere (Figure 3B). Finally, Inter-hemispheric connectivity was obtained by averaging PLV values across the connections between the 6 mirrored electrodes in the left and right hemispheres (Figure 3C).

Since EEG metrics are susceptible to individual and environmental factors, we conducted a baseline correction to PLV values at each scale. That is, for every participant, we split the 7-min recording into the first 15-second greeting phase (the baseline) and the remaining learning phase and subtracted the mean PLV in the baseline from the mean PLV in the learning phase. This way, a baseline corrected PLV (hereinafter referred to as ΔPLV) was computed per participant enabling a non-confounded comparison of brain activity changes induced by the learning task in each experimental group. In sum, for every participant, mean ΔPLV were obtained at three connectivity scales in three frequency bands which were then compared between groups using statistical tests.

### 2.5 Statistical analysis

To clarify differences between the experimental conditions, the obtained ΔPLV values and participants' scores on the word recall test were compared between the two Robot and Display groups. For all variables a group comparison was conducted using either a student's *t*-test or the non-parametric equivalent Mann-Whitney U test following the Shapiro-Wilk test of normality. The only exception was for the Intra-hemispheric ΔPLV values where two factors needed to be considered; Group (between-subjects factor: Robot vs. Display) and Hemisphere (within-subjects factor: Left vs. Right). For this variable, a 2 × 2 mixed factorial ANOVA was employed to determine the impact of experimental condition and target hemisphere on connectivity changes. Where the assumption of normality was violated, we used permutation-based ANOVA (*N* = 10,000 simulations) using Manly's approach of unrestricted permutations (Manly, 2006).

To examine the relationship between functional connectivity in each frequency band and the word recall scores, a Multiple Linear Regression (MLR) analysis was employed to model the relationship using four independent variables (IV); three continuous variables (Global ΔPLV in theta, alpha and beta frequency bands) and one categorical variable (experimental groups with Display group as the reference). To assess the goodness-of-fit of the model and its respective predictors, the full model (consisting of all IVs) was compared to four reduced models, each excluding one IV at a time. The coefficient of determination (*R*^2^) and the adjusted *R*^2^ are reported as a measure of variability explained and the variable's added value to the model. Additionally, to assess the predictive quality of the model, Cross-Validation using Leave-One-Out (LOOCV) was conducted and the Root Mean Squared Error (RMSE) was obtained. ANOVA tests were then conducted to examine whether the full model significantly differed in the sum of squared residuals (SSR) from the reduced models. The full model's regression can be defined as Equation 2:


(2) $$\begin{array}{l} y=\beta_{0}+{\beta_{1}X}_{1}+\beta_{2}X_{2}+\beta_{3}X_{3}+\beta_{4}X_{4}+\varepsilon \end{array}$$

where *y* is the target variable “word recall test score”, *X*_1_, *X*_2_, *X*_3_ and *X*_4_ are the predictor variables, β_0_, β_1_, β_2_, β_3_, β_4_ and ε are the coefficients and the error term, respectively.

## 3 Results

### 3.1 Global functional connectivity

**Figure 4:**
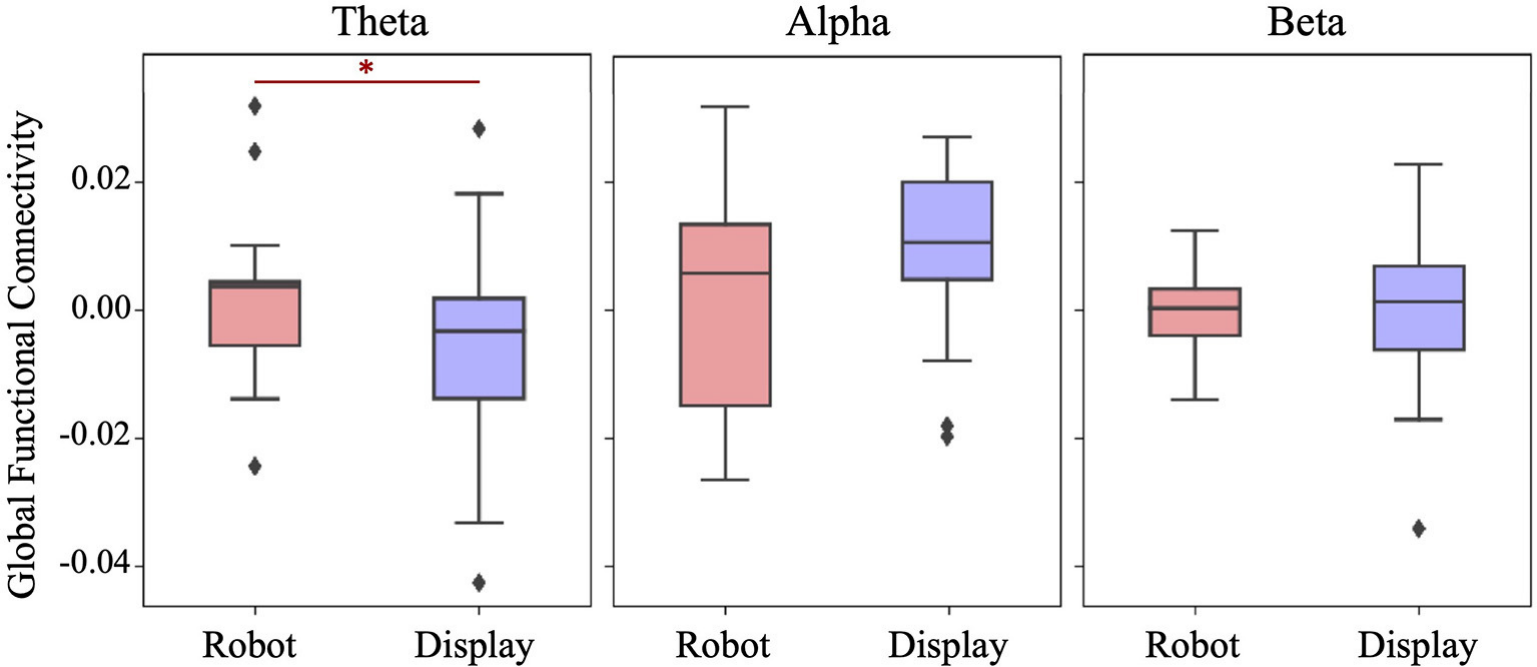
Boxplots showing the Global functional connectivity per frequency band in each group. A significantly higher Global FC in the theta band was observed in the Robot group. *p < 0.05.

Figure 4 shows the distribution of Global functional connectivity obtained in three frequency bands for each experimental group. Global FC was obtained by averaging ΔPLV values from all possible 66 connections across 12 EEG electrodes. A one-tailed student's *t*-test indicated that the Robot group had significantly higher theta band synchronization than the Display group (*t*_(39)_ = 1.798, *p* = 0.04). No significant differences were found between the groups with respect to the alpha band (*t*_(39)_ = −1.407, *p* = 0.916) and beta band (*t*_(39)_ = 0.153, *p* = 0.44) connectivity.

### 3.2 Intra-hemispheric functional connectivity

To examine Intra-hemispheric FC between groups, mixed (2 × 2) ANOVAs were used to test the main effect of experimental Group (Robot vs. Display) and target Hemisphere (Right vs. Left) on intra-hemispheric ΔPLV values in each frequency band. The results are summarized in Table 1. As can be seen in this table, no significant main effect and subsequently no interaction was observed between variables in any of the frequency bands.

**Table 1:** Mixed ANOVA results for Intra-hemispheric functional connectivity per frequency band.

	**Sum sq**	**Df**	**F**	***P*-value**
**Theta (*****N*** = **10,000)**				
Group	5 × 10^−4^	1	1.518	0.223
Hemisphere (L/R)	1 × 10^−4^	1	0.394	0.535
Group:Hemisphere	9 × 10^−4^	1	2.444	0.123
Residual	0.0278	78	–	–
**Alpha**				
Group	0.0039	1	2.563	0.113
Hemisphere (L/R)	19 × 10^−6^	1	0.041	0.841
Group:Hemisphere	75 × 10^−6^	1	0.158	0.692
Residual	0.0371	78	–	–
**Beta (*****N*** = **10,000)**				
Group	1 × 10^−4^	1	5.351	0.591
Hemisphere (L/R)	4 × 10^−4^	1	0.163	0.129
Group:Hemisphere	3 × 10^−5^	1	0.301	0.685
Residual	0.1401	78	–	–

N, number of permutations; L, left; R, right; Sum sq, sum of squared residuals.

### 3.3 Inter-hemispheric functional connectivity

The Inter-hemispheric FC was computed by averaging the ΔPLV values across 6 connections between homologous EEG electrodes. The obtained mean values were compared between Robot and Display groups using a student's *t*-test for theta band and Mann-Whitney *U*-tests for alpha and beta bands due to non-normal distribution of values in these two bands. The results are summarized in Table 2. The tests indicated that Inter-hemispheric FC in the theta band was significantly larger in the Robot group (*M* = 0.003, *SD* = 0.022) than the Display group (*M* = −0.010, *SD* = 0.026), *t*_(39)_ = 1.722, *p* = 0.047. However, no significant difference was observed in the alpha band (*Mdn*_*Robot*_ = −0.001, *Mdn*_*Display*_ = −0.019, *U*(39) = 151, *p* = 0.939), and the difference in the beta band was only marginally significant (*Mdn*_*Robot*_ = 0.003, *Mdn*_*Display*_ = −0.004, *U*(39) = 273, *p* = 0.052).

**Table 2:** Comparison of inter-hemispheric functional connectivity between robot and display group in each frequency band.

**Frequency band**	***T/U* statistic**	***p*-value**	**Statistical test**
Theta	**1.722**	**0.047** ^*^	Student's *t*-test
Alpha	151	0.939	Mann-Whitney U
Beta	273	0.052	Mann-Whitney U

Significant comparisons are marked in bold with ^*^.

### 3.4 Correlation between brain activity and language learning

Children's learning performance on the post-interaction word recall test was slightly higher in the Robot group (*mdn* = 0.839) as compared to the Display group (*mdn* = 0.708), however this difference was not significantly different (*U* = 146, *p* = 0.07).

To assess the relationship between group's learning performance and their brain activity, a Multiple Linear Regression analysis was calculated to predict children' performance on the word recall test based on 4 variables (i.e., Global FC in theta, alpha and beta bands and the experimental group). All assumptions for a Multiple Linear Regression were met. Since no significant interaction was observed between group and ΔPLV in any of the frequency bands, the interaction terms were excluded from the analysis. In addition to the full model, four reduced models each excluding one predictor were regressed and their goodness-of-fit was compared to the full model using RMSE as error measure and *R*^2^ as the measure of the model's contribution to prediction of test scores.

The summary results of the models are provided in Tables 3, 4. A significant regression equation was found for the model excluding alpha band functional connectivity (*F*_(3,28)_ = 6.429, *p* = 0.002), with adjusted *R*^2^ of 0.344. Participant's predicted word test score was equal to 0.714 + 3.391 (theta FC) − 5.105 (beta FC) + 0.115 (group), where group was coded as 1 = Robot and 0 = Display (see Table 5). The Robot group scored 11.5% higher on average than the Display group. In sum, functional connectivity in the theta band and experimental group were significant predictors of the children's scores in the word recall test.

**Table 3:** Model summary comparison.

**Model**	**SSR**	**R^2^**	**R^2^ adj**.	**Df (res.)**	**Df (mod.)**	**RMSE**
Full model	0.463	0.413	0.326	27	4	0.144
Excluding theta	0.544	0.31	0.236	28	3	0.152
Excluding alpha	**0.467**	**0.408**	**0.344**	**28**	**3**	**0.137**
Excluding beta	0.504	0.362	0.293	28	3	0.145
Excluding group	0.544	0.31	0.236	30	1	0.148

The best-fit model is the model that includes group, theta and beta functional connectivity (marked in bold).

SSQ, Sum of Squared Residuals; adj., Adjusted; Df, degrees of freedom; res., residuals; mod., model; RMSE, Root Mean Squared Error.

**Table 4:** ANOVA results comparing residuals between full model and reduced models.

**Model**	**SSR**	**Df**	**ΔSS**	***F***	***p***
Full model	0.463	28	–	–	–
Theta	**0.544**	**27**	**−0.082**	**4.76**	**0.038** ^*^
Alpha	0.467	27	−0.004	0.241	0.628
Beta	0.504	27	−0.041	2.373	0.135
Group	**0.544**	**27**	**−0.081**	**4.738**	**0.038** ^*^

Significant predictors that were removed from the reduced models are marked in bold with ^*^.

**Table 5:** Output of the regression for the best-fit models.

**Effect**	**Estimate**	**SE**	**95% CI**		***p***
			**LL**	**UL**	
Intercept	0.714	0.033	0.645	0.782	0.000
Theta	3.391	1.572	0.170	6.611	0.040
Beta	−5.105	3.014	–11.278	1.068	0.101
Group	0.115	0.048	0.017	0.212	0.023

Degrees of freedom (residuals)= 28, degrees of freedom (model)= 3. CI, confidence interval; LL, lower limit; UL, upper limit.

## 4 Discussion

The aim of the current study was to explore the effect of robot-assisted language learning (RALL) on children' brain activity and to elucidate the relationship between children's brain responses during the learning phase with their learning performance afterwards. EEG activity was collected from two groups of children who learned a new language either from an embodied social robot or using a computer screen. To assess the impact of the used technology on children's brain responses, changes in EEG functional connectivity (FC) was computed as a measure of communication between different brain regions, which has been previously associated with language learning and development (Gaudet et al., 2020). Results indicated that children who learned a new language from a social robot demonstrated a significantly larger change of FC in the theta frequency band, particularly across inter-hemispheric connections (between electrodes of the left and right hemispheres). Moreover, children's learning gain, as measured through a post-interaction word test, could be predicted by their theta band FC and the experimental group they were assigned to, with children in the Robot group achieving a higher score in the test.

These findings are consistent with previous neuroscientific evidence that highlight the importance of theta frequency band in language processing and development in children and adolescents (Meyer et al., 2019). Several studies have already indicated the prominent role of theta-band connectivity in language learning (Doesburg et al., 2016) and production (Ewald et al., 2012). Additionally, theta-band connectivity is known to play an essential role in healthy language development and memory retrieval (Meyer, 2018; Gaudet et al., 2020). Such findings are congruent with our observation of significant synchronization of EEG signals across distributed brain regions in the theta frequency band and its significant relationship with better word learning (as indicated by the MLR analysis) particularly for the children who interacted with a social robot.

On the other hand, the results regarding stronger theta band inter-hemispheric connectivity in the Robot group are of interest as they challenge the traditional view that the left hemisphere primarily houses the neural signatures of language learning (Qi and Legault, 2020). Recent studies provide evidence that both hemispheres contribute equally to second language processing (Gaudet et al., 2020) and that inter-hemispheric connectivity is implicated in second language acquisition particularly in early stages of learning (Sander et al., 2023). Based on these findings, we can argue that the children group who learned a new language from the robot experienced a stronger activation of brain networks that have been previously associated with language development and learning.

The findings of this study provide, for the first time, a neuroscientifically grounded evidence for the effectiveness of social robots in facilitating second language learning. Previous research has constantly argued for the benefit of robot tutors in child-robot interaction to support early language development, as well as second language acquisition (Kennedy et al., 2016; Randall, 2019; Van den Berghe et al., 2019; Vogt et al., 2019). These studies often argue that the physical presence (embodiment) of a robot as well as some of its anthropomorphic features and behavior can improve the child engagement and tutoring outcomes (Alimardani et al., 2021) because children do not learn language just by listening and association; rather, through social interaction (Belpaeme et al., 2018b; Li and Jeong, 2020).

The design of the current study entails limitations that should be considered when interpreting the results. First, the study employed a between-subjects design which could have impacted the outcomes due to the inherent individual differences that exist in both brain activity and baseline language learning skills of children. While we tried to counter this issue by incorporating a baseline-correction in the EEG analysis, future research should try to minimize the impact of individual differences by considering within-subjects design, where for example the same child is exposed to both technology forms in two different learning sessions. Additionally, the children in the Robot group might have been affected by the novelty effect (Belpaeme, 2020), causing a stronger brain response due to their first-time exposure to a social robot. Hence, it is encouraged that future research examines the validity of these findings when the RALL interaction is repeated over multiple sessions.

The application of neurophysiological methods as an objective measure of human-robot interaction (HRI) is quite scarce in the literature. Previous research has only produced a small number of studies that highlight the benefits of neuroimaging tools such as EEG for objective assessment of user experience during interaction with a social robot (Alimardani et al., 2020, 2021, 2022; Roy et al., 2020; Yoon et al., 2021). Particularly, in the context of child-robot interaction and evaluation of pedagogical robots, the children may not be able to reliably answer surveys and their learning success could be dependent on various internal and external factors that are not directly measurable or controlled for when employing behavioral metrics (Belpaeme et al., 2013; Nakov and Alimardani, 2022). In such scenarios, the study of neurophysiological responses via wearable sensing technology could provide a more reliable metric of robot's impact on children's interaction and learning processes (Leite et al., 2013; Alimardani et al., 2021). Additionally, neurophysiological measurements allow for real-time monitoring and adaptation of robot behavior in order to maintain a learner's attention and engagement (Alimardani and Hiraki, 2020; Prinsen et al., 2022; Vrins et al., 2022). Thus, the outcome of this study on validating EEG brain activity measures associated with language learning (such as FC) offers promising opportunities for future research to design robot tutors that can personalize timing, feedback strategies, and lesson content for each individual learner using their neurophysiological data.

## Data availability statement

The raw data supporting the conclusions of this article will be made available by the authors, without undue reservation.

## Ethics statement

The studies involving humans were approved by Ethics Committee of the University of Tokyo. The studies were conducted in accordance with the local legislation and institutional requirements. Written informed consent for participation in this study was provided by the participants' legal guardians/next of kin.

## Author contributions

MA: Conceptualization, Methodology, Supervision, Writing—original draft, Writing—review & editing. JD: Formal Analysis, Methodology, Visualization, Writing—review & editing. A-LJ: Conceptualization, Funding acquisition, Methodology, Project administration, Supervision, Writing—review & editing. KH: Conceptualization, Funding acquisition, Methodology, Project administration, Supervision, Writing—review & editing.
